# IRF8 aggravates nonalcoholic fatty liver disease via BMAL1/PPARγ axis

**DOI:** 10.1016/j.gendis.2024.101333

**Published:** 2024-05-20

**Authors:** Xinyue Li, Hong Zhang, Fan Yu, Shuting Xie, Tongyu Wang, Rong Zhang, Guangzhong Xu, Liang Wang, Yeping Huang, Cheng Hu

**Affiliations:** a Shanghai Diabetes Institute, Shanghai Key Laboratory of Diabetes Mellitus, Shanghai Clinical Centre for Diabetes, Shanghai Sixth People's Hospital Affiliated to Shanghai Jiao Tong University School of Medicine, Shanghai 200233, China; b Institute for Metabolic Disease, Fengxian Central Hospital Affiliated to Southern Medical University, Shanghai 201406, China; c Surgery Centre of Diabetes Mellitus, Capital Medical University Affiliated Beijing Shijitan Hospital, Beijing 100038, China

**Keywords:** Brain and muscle ARNT-Like 1, Hepatic steatosis, Interferon regulatory factor 8, Lipogenesis, Non-alcoholic fatty liver disease, Peroxisome proliferator-activated receptor γ, Transcriptional regulation

## Abstract

Non-alcoholic fatty liver disease (NAFLD) is a hepatic metabolic syndrome arising from lipid metabolic imbalance, with its prevalence increasing globally. In this study, we observed a significant up-regulation of interferon regulatory factor 8 (IRF8) in the liver of NAFLD model mice and patients. Overexpression of IRF8 induced lipid accumulation in the mouse primary hepatocytes. Mice with adeno-associated virus-mediated IRF8 overexpression exhibited hepatic steatosis due to up-regulated peroxisome proliferator-activated receptor γ (PPARγ) expression and increased fatty acid uptake and lipogenesis. *In vitro*, small interfering RNA-mediated IRF8 knockdown attenuated triglyceride accumulation by dampening PPARγ expression through transcriptional inhibition of brain and muscle ARNT-like 1. The PPARγ-specific antagonist GW9662 abolished the effect of IRF8 overexpression. Furthermore, adeno-associated virus-mediated IRF8 knockdown in the mouse liver markedly alleviated hepatic steatosis and obesity-related metabolic syndrome. These findings indicate that IRF8 plays a vital role in modulating hepatic lipid metabolism in a PPARγ-dependent manner and provide a previously unknown insight into NAFLD therapeutic strategies.

## Introduction

Non-alcoholic fatty liver disease (NAFLD) represents a pathological process ranging from hepatic steatosis due to excessive lipid accumulation in hepatocytes to non-alcoholic steatohepatitis with fibrosis, cirrhosis, and hepatocellular carcinoma.^1^^,^^2^ Hepatic steatosis is correlated with the dysregulation of systemic lipid and glucose homeostasis.^3^ Typically, hepatic steatosis arises from the abnormal accumulation of triglyceride (TG) within the liver, stemming from a disruption in the equilibrium between lipogenesis and fatty acid oxidation.^4^

Interferon regulatory factor 8 (IRF8), initially identified as interferon consensus sequence binding protein (ICSBP),^5^ belongs to the interferon regulatory factor superfamily. IRF8 is known for its roles in interferon γ inducibility and the regulation of immune cell differentiation, non-hematopoietic cell proliferation, and the pathogenesis of tumors.^6^, ^7^, ^8^, ^9^, ^10^ While IRFs are traditionally known for their immunoregulatory roles, emerging evidence has identified their involvement in controlling energy metabolism. Hepatic IRF3 aggravates dysglycemia in obesity.^11^ IRF6 alleviates liver inflammation and metabolic disorders in mice through transcriptional suppression of peroxisome proliferator-activated receptor γ (PPARγ).^12^ IRF7 exerts a protective role against diet-induced obesity and insulin resistance.^13^ These findings suggest a potential role of the IRF family in modulating metabolism and energy homeostasis. A previous study reported that IRF8 promotes the phosphorylation of the inflammatory regulator NLRC4 (nucleotide-binding domain leucine-rich repeat CARD domain containing 4), leading to macrophage senescence.^14^ However, the role of IRF8 in regulating glucose homeostasis and lipid metabolism remains obscure.

In this study, we elucidate the role of IRF8 in hepatic lipid metabolism using mouse models of gain and loss of function. Through *in vivo* and *in vitro* experiments, we provide evidence that hepatic IRF8 fosters lipid accumulation by positively regulating the transcription of brain and muscle ARNT-like 1 (BMAL1), subsequently increasing PPARγ expression and consequent hepatic steatosis, contributing to the onset of NAFLD. We provide a previously unknown insight into IRF8-mediated maintenance of hepatic lipid homeostasis. This discovery may offer a potential therapeutic target for NAFLD.

## Material and methods

### Human liver

All human liver tissues were obtained from the Capital Medical University Affiliated Shijitan Hospital. All participants involved in the study signed informed consent forms. All procedures complied with the Helsinki Declaration and were approved by the Ethics Committee of the Capital Medical University Affiliated Shijitan Hospital [ethics approval number: sjtky11-1x-2022(098)].

### Animal studies

Eight-week-old C57BL/6J mice were purchased from GemPharmatech Company (Nanjing, China). The mice were randomly allocated into two groups and fed with a chow diet (CD) (10 kcal of fat, 70 kcal of carbohydrates, and 20 kcal of proteins, P1200F-25, Shanghai Puluteng, China) or high-fat diet (HFD) (60 kcal of carbohydrates and 20 kcal of proteins, D12492, Research Diets, USA) for 20 weeks to create diet-induced NAFLD model mice. The non-alcoholic steatohepatitis model mice were fed with a diet containing 40 kcal of fat, 20 kcal of fructose, and 2% cholesterol (D09100310, Research Diets) for 24 weeks. For the construction of the IRF8 knockdown model, 8-week-old C57BL/6J mice were given adeno-associated virus (AAV)-shIRF8 or AAV-green fluorescent protein (GFP) via tail vein injection with a titer of 1 × 10^10^ vg. The AAV-injected mice were fed with a CD or HFD diet for 12 weeks. For the construction of the IRF8 overexpression model, 8-week-old C57BL/6J mice were given AAV-IRF8 or AAV-GFP via tail vein injection with a titer of 1 × 10^10^ vg. AAV was purchased from Obio Zhizao Gene Technology Company (Shanghai, China). Knockdown virus sequences can be accessed in Table S1. All mice were housed in the animal facility of the Sixth People's Hospital Affiliated to the Shanghai Jiao Tong University School of Medicine, where they had *ad libitum* access to water and food. All animal experimental procedures were approved by the Ethics Committee of the Sixth People's Hospital Affiliated to the Shanghai Jiao Tong University School of Medicine.

### Mouse primary hepatocyte extraction and transfection

Mouse primary hepatocytes (MPHs) were isolated from the livers through portal vein injection of collagenase IV (C5138, Sigma–Aldrich, USA), following previously described protocols by Charni-Natan et al.^15^ Eight-week-old C57BL/6J male mice were anesthetized with isoflurane (RWD Life Science, China). The liver was perfused from the inferior vena cava with a pre-heated (37 °C) calcium-free solution (containing 8.3 g NaCl, 0.5 g KCl, 2.4 g HEPES, and 0.015 g EDTA per liter of solution) until the liver was devoid of blood. Subsequently, a solution of type IV collagenase heated to 37 °C was perfused into the liver. The perfusion was stopped when the liver collapsed and cracks appeared on its surface. The liver was then quickly excised and in cold Dulbecco's modified Eagle's medium (DMEM, 11965092, Gibco, USA) containing 5% fetal bovine serum to release the isolated hepatocytes. The obtained cell suspension, after being centrifuged at 40 *g* for 1 min, was re-suspended in cold DMEM, and the cell pellet was filtered through a 70-μm cell strainer, followed by another centrifugation at 40 *g f*or 1 min. The supernatant was discarded to obtain the hepatocyte pellet (MPHs). The pellet was re-suspended in a mixture of 5 mL DMEM, 4.5 mL Percoll (17089102, Cytiva, USA), and 0.5 mL 10× phosphate-buffered saline, and centrifuged at 80 *g* for 10 min to obtain purified MPHs. The purified MPHs were then re-suspended in DMEM supplemented with 10% fetal bovine serum and 1% penicillin-streptomycin, seeded in 12-well plates at a density of 5 × 10^5^ cells/well. To mimic fatty liver *in vitro*, MPHs were incubated with palmitic acid (200 μM) for 24 h. Adenovirus vectors expressing IRF8 (Ad-IRF8) were transfected for 48 h to induce IRF8 overexpression, while an adenovirus expressing GFP (Ad-GFP) was used as control. All adenoviruses were purchased from Obio Zhizao Gene Technology Company (Shanghai, China) and transfected at a titer of 5 × 10^7^ vg/mL. Small interfering RNAs (si-IRF8 and si-BMAL1) were respectively utilized for gene silencing of IRF8 and BMAL1 over 48 h, with a non-specific small interfering RNA (si-NC) serving as the silencing control. All small interfering RNAs were purchased from Genepharma Company (Suzhou, China) and used at a dosage of 60 pmol. MPHs were treated with the PPARγ antagonist GW9662 (10 μM) (S2915, Selleck, USA) to inhibit PPARγ for 24 h. All si-RNA sequences can be accessed in Table S2.

### RNA sequencing

The MPHs were used to extract total RNA with the Universal RNA Extraction CZ Kit (RNC643, ONREW, China) following the manufacturer's instructions. The RNA quantity was measured using Qubit 4.0 (Invitrogen, USA), and quality was assessed by denaturing agarose gel electrophoresis. RNA libraries were prepared using the VVAHTS® Universal V8 RNA-seq Library Preparation Kit for Illumina (NR605-0, Vazyme, China) and sequenced using the Illumina NovaSeq 6000 platform with a 150 paired-end sequencing strategy. Skewer v0.2.2 was used to process the raw data, and FastQC v0.11.2 was used to check data quality. The read length was 2 × 150 bp. The clean reads were aligned to the mouse genome (mm10) in ensemble using STAR, allowing for one mismatch. Gene expression data was generated using StringTie (v1.3.1c), and differential gene expression was analyzed with DESeq2 (v1.16.1). The thresholds for determining differentially expressed genes were set at *p* < 0.05 and absolute fold change ≥2.

### Western blots

The liver tissues and hepatocytes were homogenized and lysed in RIPA buffer (50 mM Tris–HCl, pH 7.4, 150 mM NaCl, 1 mM EDTA, 1% Triton X-100, 0.5% sodium deoxycholate, 0.1% SDS) (P0013B, Beyotime Biotechnology, China) supplemented with protease and phosphatase inhibitors (Sigma–Aldrich). The protein concentration was determined using the bicinchoninic acid (BCA) assay (P0011, Beyotime Biotechnology, China). Equal amounts of proteins were separated using SDS-PAGE gel and then electrophoretically transferred to a 0.22 μm NC membrane (Millipore, USA). The membrane was blocked with 5% bovine serum albumin at room temperature for 1 h. The specific primary antibody was incubated overnight at 4 °C. Following primary antibody incubation, the membrane was washed three times with phosphate-buffered saline with 0.1% Tween® 20 detergent for 5 min each time and then incubated with horseradish peroxidase-conjugated secondary antibody (1:2500 dilution in blocking solution) at room temperature for 1 h. After three washes with phosphate-buffered saline with 0.1% Tween® 20 detergent, chemiluminescence was detected using developing reagents (Millipore, USA). Digital imaging systems (Thermo Fisher Scientific, USA) were used to acquire images. The antibodies including anti-IRF8 (1:1000; 5628S, Cell Signaling Technology, USA), anti-BMAL1 (1:1000; 14020S, Cell Signaling Technology), anti-PPARγ (1:1000; 5628S, Cell Signaling Technology), anti-cluster of differentiation 36 (CD36; 1:1000; ab133625, Abcam, UK), anti-stearoyl-CoA desaturase 1 (SCD1; 1:1000; 2794S, Cell Signaling Technology), anti-sterol regulatory element-binding protein 1c (SREBP1-1c; 1:500; sc-365513, Santa Cruz Biotechnology, USA), anti-HSP90 (1:1000; sc-101494; Santa Cruz Biotechnology), anti-Mouse (1:5000; 7076S, Cell Signaling Technology), and anti-Rabbit (1:5000; 7074S, Cell Signaling Technology) were diluted in 5% bovine serum albumin.

### Real-time quantitative PCR

RNA was extracted from tissues or cells using Trizol (15596026, Thermo Fisher Scientific, USA). The concentration of RNA was quantified using a NanoDrop spectrophotometer (Thermo Fisher Scientific, USA). Total RNA was transcribed into cDNA using a TaKaRa kit (RR036A, TaKaRa, China). Quantitative PCR (qPCR) was performed on a Light Cycler 480 (Roche, Switzerland) using Fast SYBR Green Master Mix (A25742, ABI, USA). Relative gene expression levels were calculated using the 2^−ΔΔCt^ method and normalized to the expression level of the housekeeping gene *Actb*. The results were expressed as fold change relative to the control. The primers used in the experiments are shown in Table S3.

### Hematoxylin and eosin (H&E) staining

The paraffin sections were subsequently immersed in environmentally friendly dewaxing solution Ⅰ for 20 min, environmentally friendly dewaxing solution Ⅱ for 20 min, anhydrous ethanol Ⅰ for 5 min, anhydrous ethanol Ⅱ for 5 min, and 75% alcohol for 5 min, and washed with tap water. Sections were stained in hematoxylin staining solution for 3–5 min, washed with tap water, differentiated in differentiation solution, washed with tap water, returned to the blue solution, and rinsed with running water. Sections were sequentially dehydrated in 85% and 95% graded alcohol for 5 min each and stained in eosin for 5 min. Sections were then sequentially placed in anhydrous ethanol I for 5 min, anhydrous ethanol II for 5 min, anhydrous ethanol III for 5 min, dimethyl I for 5 min, and dimethylbenzene II for 5 min for transparency, and sealed with neutral gum.

### Oil Red O staining

Oil Red O stock solution (O0625, Sigma–Aldrich) was prepared by combining the dye with distilled water at a 3:2 (v/v) ratio. This mixture was refrigerated at 4 °C overnight, filtered the following day, and stored at 4 °C for additional incubation before a second filtration to yield the working solution. Hepatic sections underwent Oil Red O staining in darkness for 8–10 min, brief air exposure, differentiation in 60% isopropyl alcohol baths, and sequential rinsing in purified water. After hematoxylin counterstaining and additional rinsing, sections were mounted with glycerol gelatin. For MPHs, following paraformaldehyde fixation and phosphate-buffered saline washes, Oil Red O incubation was performed for 15 min, with subsequent phosphate-buffered saline rinsing before microscopy. Lipid quantification involved Oil Red O solubilization with 60% isopropyl alcohol and absorbance measurement at an optical density of 510 nm.

### Intraperitoneal injection glucose tolerance test (IPGTT) and intraperitoneal injection insulin tolerance test (IPITT)

The mice underwent fasting for 12 h or 4 h before IPGTT or IPITT. Baseline blood samples were collected using blood glucose test strips (06454038, Roche) from the tail vein to determine fasting glucose levels. Intraperitoneal injections of glucose solution (2 g/kg body weight) for IPGTT or insulin solution (1.5 IU/kg body weight) were administered. Plasma glucose was measured at 15, 30, 60, 90, and 120 min after glucose ingestion. Data were plotted as glucose concentration versus time to visualize the glucose curve. Areas under the curve were calculated by the trapezoidal rule to quantify glucose tolerance or insulin tolerance.

### Luciferase reporter assay

The potential transcription factor binding sites within the *Bmal1* (−2000 to 0) and *Pparγ* promoters (−2000 to 0) were analyzed using the JASPAR database (http://jaspardev.genereg.net/). Promoter sequences for wild-type *Irf8* and *Bmal1* were amplified from the mouse genomic DNA and cloned into the pGL3-basic luciferase reporter vector (Sangon Biotech, China). The expression plasmids of IRF8 and BMAL1 (pcDNA3.1-*Irf8* and pcDNA3.1-*Bmal1*) were obtained from Sangon Biotech Company. In luciferase reporter assays, the wild-type BMAL1 report vector (100 ng), IRF8 expression vector (200 ng), or the empty pcDNA3.1 vector as a control were co-transfected into HEK-293T cells in 24-well plates using lipofectamine 3000 (L3000150, Invitrogen, USA). A similar approach was employed for the PPARγ reporter vector with pcDNA3.1-BMAL1. After cell culture and transfection, harvested cells were assayed for luciferase activity using the Dual-Luciferase Reporter Assay System (Promega, UK) according to the manufacturer's instructions.

### Plasma and hepatic biochemical measurements

Plasma alanine aminotransferase (ALT) and aspartate aminotransferase (AST) were quantified using assay kits (03036926, 03039631) provided by Siemens Healthcare Diagnostics Inc. For the quantification of TG in liver tissues, samples weighing between 30 and 50 mg were homogenized in 500 μL phosphate buffered saline with a micro-homogenizer. The resulting homogenate was then centrifuged at 12,000 *g* for 15 min. 50 μL of the supernatant was set aside for protein quantification. The remaining homogenate was thoroughly extracted with 1.5 mL of a chloroform-ethanol mixture (v/v, 2:1). After centrifugation at 12,000 *g* for another 15 min, the isolated organic phase was evaporated to dryness under a fume hood. The dried lipid residue was used for TG quantification utilizing kits provided by WAKO Chemicals (290-63701, Japan). Hepatocellular TG levels were assessed following cell lysis using a proprietary buffer (Kehua Biotech, Shanghai, China), subsequent incubation in a 70 °C water bath for 10 min, and centrifugation at 2000 *g* for 15 min. The supernatants were harvested for protein assays, and TG content was quantified using a TG assay kit (S0913, Dongou Diagnostics, China).

### Statistical analysis

Data are presented as mean ± standard error of the mean. Statistical analysis was performed using Prism 9.0 (GraphPad Software). An unpaired two-tailed Student's *t*-test was employed to compare two groups. For comparisons involving more than two groups with one factor, one-way ANOVA followed by Dunnett's or Bonferroni's multiple comparison tests was utilized. The enrichment of biological processes was analyzed using Fisher's exact test.

## Results

### Up-regulation of IRF8 in the liver of mice and humans with NAFLD

**Figure 1:**
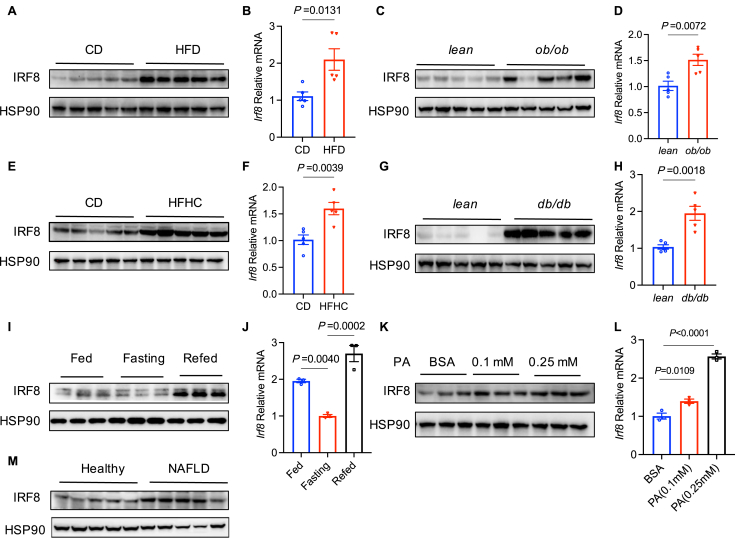
Up-regulation of IRF8 in the liver of mice and humans with NAFLD. **(A**–**H)** Western blots and quantitative PCR were performed to analyze IRF8 levels in the livers of C57BL/6J male mice fed with HFD or CD (A, B) (*n* = 5 biologically independent mice), lean or *ob/ob* male mice (C, D) (*n* = 5 biologically independent mice), C57BL/6J male mice fed with HFHC (40 kcal of fat, 20 kcal of fructose, and 2% cholesterol) or CD for 30 weeks (E, F) (*n* = 5 biologically independent mice), and lean or *db/db* male mice (G, H) (*n* = 5 biologically independent mice). **(I, J)** After culturing MPHs using DMEM plus 10% fetal bovine serum for 24 h, the relative protein and mRNA expression levels of IRF8 were measured. The remaining cells were further cultured in DMEM for another 24 h to detect the expression level of IRF8. Subsequently, the remaining cells were cultured in DMEM plus 10% fetal bovine serum for an additional 24 h to evaluate the relative protein and mRNA expression levels of IRF8. **(K, L)** MPHs were treated with 5% bovine serum albumin, 0.1 mM palmitic acid (PA), and 0.25 mM PA for 24 h, and then the mRNA (K) and protein (L) levels of IRF8 were examined. **(M)** IRF8 protein levels were analyzed in liver tissues from healthy or patients with NAFLD by western blots (*n* = 5 biologically independent individuals). Data are presented as mean ± standard error of the mean. *p* values were calculated using an unpaired two-tailed Student's *t*-test (B, D, F, H, J–L). IRF8, interferon regulatory factor 8; NAFLD, non-alcoholic fatty liver disease; HFD, high-fat diet; CD, chow diet.

To investigate the potential association between IRF8 and hepatic lipid metabolism, we utilized four murine models: leptin receptor-deficient mice (*db/db*), leptin-deficient mice (*ob/ob*), C57BL/6J mice fed an HFD, and C57BL/6J mice fed an HFD supplemented with high cholesterol and high fructose, all established models of NAFLD.^16^ Western blots and qPCR analysis indicated elevated levels of IRF8 in the liver tissues of humans and mice with NAFLD (Fig. 1A–H, M). We also examined the response of IRF8 to energy metabolic states and found that, compared with the physiological state, there was a decrease in IRF8 expression after 24 h of starvation treatment in MPHs, which was reversed upon refeeding (Fig. 1I, J). To further explore the changes of IRF8 during the lipid accumulation process, we treated MPHs with varying concentrations of palmitic acid and observed a dose-dependent increase in IRF8 expression (Fig. 1K, L). In summary, our findings demonstrate an up-regulation of IRF8 expression in the livers with NAFLD.

### Up-regulation of IRF8 promotes hepatic lipid accumulation

**Figure 2:**
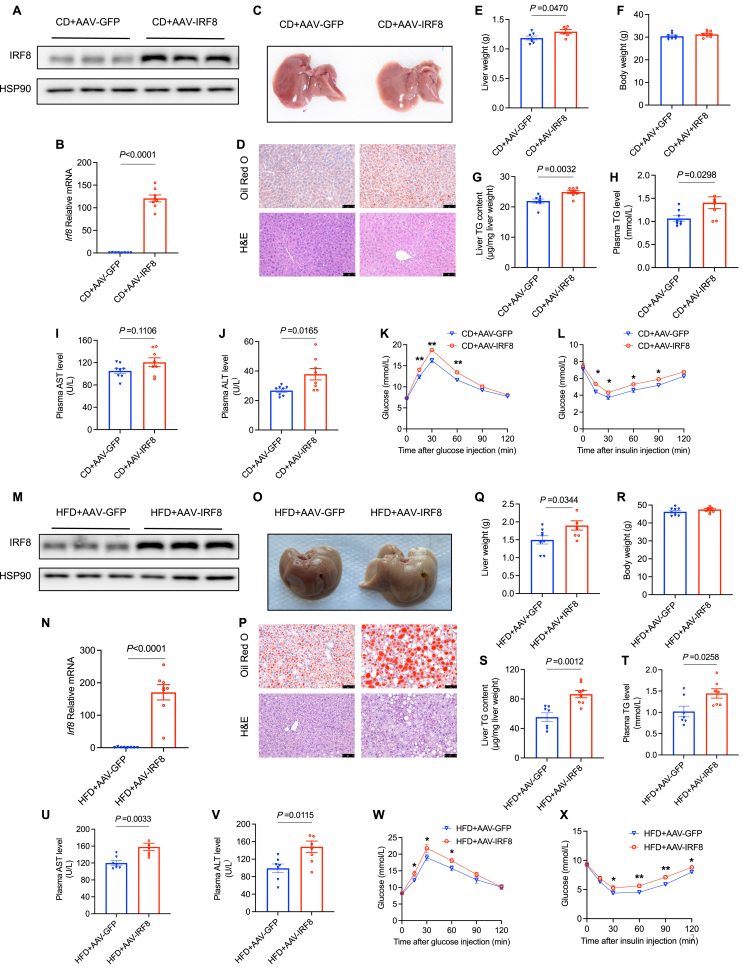
Up-regulation of IRF8 promotes hepatic lipid accumulation. **(A**–**L)** Eight-week-old C57BL/6J male mice were administered with either AAV-GFP or AAV-IRF8 via tail vein injection followed by CD-feeding for 10 weeks before being sacrificed for analysis. (A) IRF8 protein levels were examined by western blots (*n* = 3 biologically independent mice from two groups) and (B) relative *Irf8* mRNA levels were analyzed by quantitative PCR in the livers (*n* = 8 biologically independent mice from two groups). (C) The gross morphology of liver specimens. (D) Hepatic sections were stained with hematoxylin-eosin and Oil Red O. Scale bars: 75 μm. (E) Liver weight, (F) body weight, (G) hepatic and (H) plasma TG levels, (I) plasma AST, and (J) plasma ALT were analyzed (*n* = 8 biologically independent mice from two groups). (K) IPGTT and (L) IPITT were performed (*n* = 8 biologically independent mice from two groups). **(M**–**X)** Eight-week-old C57BL/6J mice received tail vein injections of either AAV-GFP or AAV-IRF8, followed by HFD-feeding for 10 weeks before being sacrificed for analysis. (M) IRF8 protein levels were examined by western blots and (N) relative *Irf8* mRNA levels were analyzed by quantitative PCR in the livers (*n* = 8 biologically independent mice from two groups). (O) The gross morphology of liver specimens. (P) Hepatic sections were stained with hematoxylin-eosin and Oil Red O. Scale bars: 75 μm. (Q) Liver weight, (R) body weight, (S) hepatic and (T) plasma TG levels, (U) plasma AST, and (V) plasma ALT were examined (*n* = 8 biologically independent mice from two groups). (W) IPGTT and (X) IPITT were performed (*n* = 8 biologically independent mice from two groups). Data are presented as mean ± standard error of the mean. *p* values were calculated using an unpaired two-tailed Student's *t*-test (B, E–J, N, Q–V), or by two-way ANOVA with Sidak's multiple comparisons test (K, L, W–X). IRF8, interferon regulatory factor 8; TG, triglyceride; IPGTT, intraperitoneal injection glucose tolerance test; IPITT, intraperitoneal injection insulin tolerance test; HFD, high-fat diet; CD, chow diet; AAV, adeno-associated virus; GFP, green fluorescent protein.

Upon observing IRF8 up-regulation in NAFLD contexts, we considered whether it might modulate hepatic lipid metabolism. Thus, we administered a liver-specific AAV serotype 9 vector expressing IRF8 via the thyroxine-binding globulin promoter to male C57BL/6J mice through tail vein injection (AAV-IRF8). The control group received an injection of AAV-GFP via the tail vein. To explore the influence of IRF8 on hepatic lipid metabolism under both physiological and pathological conditions, we exposed AAV-injected C57BL/6J mice to HFD or CD. Compared with the AAV-GFP group, western blots and qPCR analyses indicated a substantial elevation of hepatic IRF8 in AAV-IRF8-treated mice (Fig. 2A, B, M, N). In CD-fed mice, although body weight discrepancies were minimal, AAV-IRF8 treatment led to increased lipid accumulation, as evidenced by liver morphology (Fig. 2C), Oil Red O staining, and H&E staining (Fig. 2D). Additionally, except for body weight (Fig. 2F), AAV-IRF8 mice showed significant increases in liver weight (Fig. 2E), hepatic TG content (Fig. 2G), plasma TG (Fig. 2H), AST (Fig. 2I), and ALT (Fig. 2J) levels compared with controls. IPGTT (Fig. 2K) and IPITT (Fig. 2L) revealed that IRF8 overexpression impaired glucose tolerance and insulin sensitivity. In line with observations from the CD diet model, though no body weight difference was observed (Fig. 2R), the mice with IRF8 overexpression fed with HFD demonstrated similar metabolic damages, such as increased liver size (Fig. 2O), exacerbated hepatic steatosis (Fig. 2P), augmented liver weight (Fig. 2Q), higher levels of hepatic TG content (Fig. 2S), as well as higher plasma TG (Fig. 2T), AST (Fig. 2U), and ALT (Fig. 2V) levels. AAV-IRF8 mice also exhibited more severe impairments both in glucose (Fig. 2W) and insulin tolerance (Fig. 2X). Overall, the data suggest that IRF8 exacerbates hepatic lipid accumulation and metabolic disturbance under both standard and high-fat dietary conditions.

### IRF8 silencing inhibits hepatic lipid accumulation under an HFD

**Figure 3:**
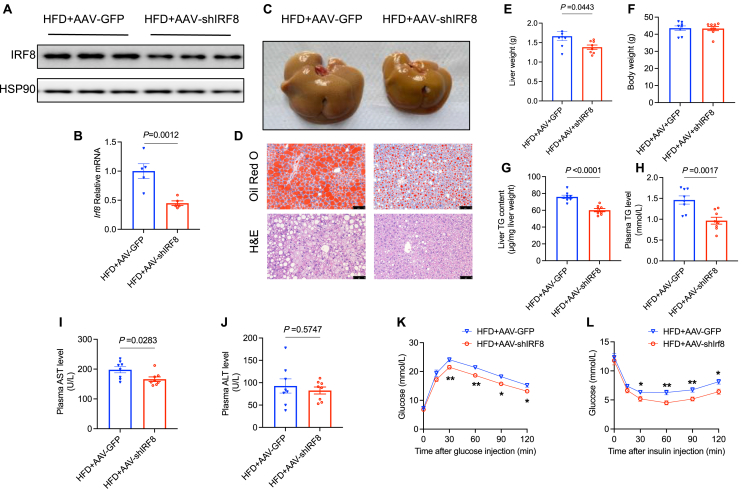
IRF8 silencing inhibits hepatic lipid accumulation under a high-fat diet. Eight-week-old C57BL/6J male mice were administered with either AAV-GFP or AAV-shIRF8 via tail vein injection followed by HFD-feeding for 10 weeks before being sacrificed for analysis. **(A, B)** IRF8 protein levels (A) were examined by western blots, and relative *Irf8* mRNA levels (B) were analyzed by quantitative PCR in the livers (*n* = 6 biologically independent mice from two groups). **(C)** The gross morphology of liver specimens. **(D)** Hepatic sections were stained with hematoxylin-eosin and Oil Red O. Scale bars: 75 μm. **(E**–**J)** Liver weight (E), body weight (F), hepatic (G) and plasma (H) TG levels, plasma AST levels (I), and plasma ALT levels (J) were examined (*n* = 8 biologically independent mice from two groups). **(K, L)** IPGTT (K) and IPITT (L) were performed (*n* = 8 biologically independent mice from two groups). Data are presented as mean ± standard error of the mean. *p* values were calculated using an unpaired two-tailed Student's *t*-test (B, E–J) or by two-way ANOVA with Sidak's multiple comparisons test (K, L). IRF8, interferon regulatory factor 8; TG, triglyceride; HFD, high-fat diet; AAV, adeno-associated virus; GFP, green fluorescent protein.

To explore the metabolic phenotype of liver-specific IRF8-knockdown mice fed an HFD, we generated liver-specific AAV9 to interfere with IRF8 expression through tail vein injection in C57BL/6J male mice. Western blots and qPCR confirmed the silencing of IRF8 (Fig. 3A, B). Compared with mice treated with AAV-GFP, those treated with AAV-shIRF8 exhibited reduced lipid accumulation in the liver, as observed by liver morphology (Fig. 3C), H&E staining, and Oil Red O staining (Fig. 3D). Furthermore, except for body weight (Fig. 3F), the AAV-shIRF8 mice showed decreased liver weight (Fig. 3E), as well as significantly reduced levels of hepatic TG (Fig. 3G), and plasma TG (Fig. 3H), AST (Fig. 3I), and ALT levels (Fig. 3J). Consistent with the mitigation of hepatic steatosis, glucose tolerance also improved after interfering with IRF8, as shown by IPGTT (Fig. 3K). Insulin resistance was alleviated as indicated by IPITT (Fig. 3L). In summary, IRF8 knockdown reduces hepatic lipid accumulation and alleviates metabolic disorders induced by an HFD.

### IRF8 transcription activates BMAL1 expression

**Figure 4:**
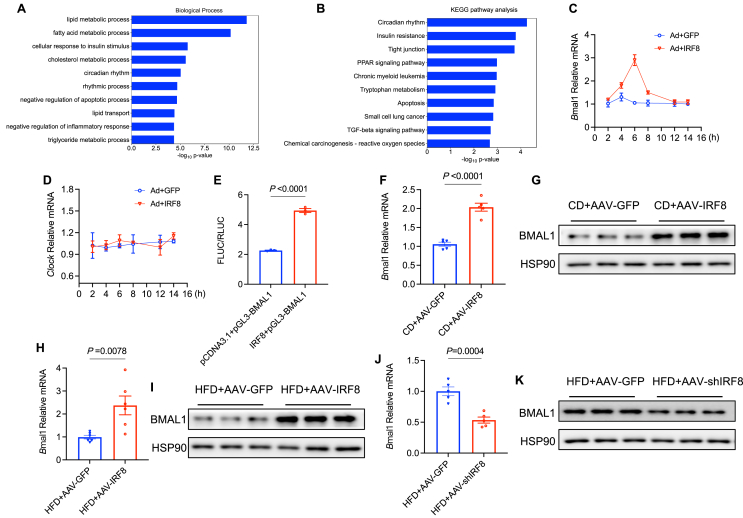
IRF8 transcription activates the expression of BMAL1. **(A, B)** Genes significantly up-regulated by Ad-IRF8 relative to Ad-GFP were cross-referenced with those significantly down-regulated by si-IRF8 as compared with si-NC to form a subset of consistently altered genes. Conversely, genes significantly down-regulated by Ad-IRF8 as compared with Ad-GFP were intersected with those up-regulated by si-IRF8 versus si-NC to form a profile of notably suppressed genes. (A) Biological process enrichment analysis and (B) KEGG pathway enrichment analysis for these intersecting genes were conducted employing Fisher's exact test. **(C, D)** Quantitative PCR was performed to analyze the relative mRNA expression of *Bmal1* (C) and *Clock* (D) after treatment with Ad-GFP or Ad-IRF8 in MPHs at the indicated time points. **(E)** HEK293T cells co-transfected with *Bmal1* promoter reporter (pGL3-*Bmal1*), either pCDNA3.1 or IRF8 overexpression plasmid, were analyzed for relative luciferase activity (FLUC/RLUC) (*n* = 3 biologically independent samples). **(F, G)** Relative mRNA levels (F) (*n* = 5 biologically independent mice from two groups) and protein levels (G) (*n* = 3 biologically independent mice from two groups) of *Bmal1* in liver tissues were measured in C57BL/6J male mice treated with AAV-IRF8 or AAV-GFP fed on CD. **(H, I)** Relative mRNA levels (H) (*n* = 5 biologically independent mice from two groups) and protein levels (I) (*n* = 3 biologically independent mice from two groups) of *Bmal1* in liver tissues were measured in C57BL/6J male mice treated with AAV-IRF8 or AAV-GFP on HFD. **(J, K)** Relative mRNA levels (J) (*n* = 5 biologically independent mice from two groups) and protein levels (K) (*n* = 3 biologically independent mice from two groups) of *Bmal1* in liver tissues were measured in C57BL/6J male mice treated with AAV-shIRF8 or AAV-GFP fed on HFD. Data are presented as mean ± standard error of the mean. *p* values for biological process and pathway enrichment were ascertained through Fisher's exact test (A, B). *p* values were calculated using an unpaired two-tailed Student's *t*-test (C–F, H, J). IRF8, interferon regulatory factor 8; BMAL1, brain and muscle ARNT-like 1; CLOCK, circadian locomotor output cycles kaput; HFD, high-fat diet; CD, chow diet; AAV, adeno-associated virus; GFP, green fluorescent protein; MPHs, mouse primary hepatocytes.

We have identified the role of IRF8 in promoting the development of NAFLD in mice. However, the underlying mechanisms of IRF8-induced lipid accumulation remain unclear. To address this, we employed Ad-IRF8 or si-IRF8 to respectively induce the overexpression or silencing of IRF8 in MPHs, using Ad-GFP or si-NC as controls. RNA sequencing was performed using hepatocytes transfected with Ad-GFP, Ad-IRF8, si-NC, and si-IRF8. Using *p* < 0.05 and fold change >2.0 as a cutoff, we identified genes that showed an opposite direction in expression pattern, including those genes up-regulated by Ad-IRF8 and down-regulated by si-IRF8, or down-regulated by Ad-IRF8 and up-regulated by si-IRF8. The resulting common differentially expressed genes were used for further analysis. Gene ontology (GO) analysis revealed a high enrichment of differentially expressed genes in fatty acid metabolism processes (Fig. 4A). Kyoto Encyclopedia of Genes and Genomes (KEGG) analysis identified an enrichment of differentially expressed genes in the circadian rhythm pathway (Fig. 4B). Circadian rhythm is an innate timing system, the core architecture of which is comprised of a transcriptional/translational negative feedback loop constituted by clock genes. Within this framework, the circadian locomotor output cycles kaput (CLOCK) and aryl hydrocarbon receptor nuclear translocator-like protein 1 (ARNTL, also known as BMAL1) serve as central master clock components of the transcriptional/translational negative feedback loop. They orchestrate 24-h oscillatory patterns by transcriptionally regulating the expression of downstream circadian genes.^17^^,^^18^ Increasing evidence suggests that many genes involved in lipid biosynthesis and fatty acid oxidation are rhythmically activated and inhibited by clock proteins. Disruption of clock function directly leads to lipid metabolism disorders, obesity, and metabolic diseases.^19^, ^20^, ^21^

Considering the consistent alterations in circadian rhythm genes observed by RNA sequencing, we hypothesize that they may be attributed to the regulation of lipid homeostasis mediated by IRF8. Previous studies reported that BMAL1 in astrocytes controls energy balance by regulating metabolic rate, hepatic and white adipose tissue lipogenesis, and brown adipose tissue activity.^22^ Furthermore, after fasting, hepatic BMAL1 promotes *de novo* lipid synthesis via the insulin-mTORC2-AKT signaling pathway.^23^ Clock mutation mice (*Clock*^mt/mt^) exhibit sustained hyperlipidemia and elevated expression of microsomal triglyceride transfer protein.^24^ We next tested our hypothesis and found that the expression of BMAL1 increased gradually and subsequently decreased throughout Ad-IRF8 treatment in MPHs (Fig. 4C), as determined by qPCR, but there was no change in the expression of CLOCK (Fig. 4D). Considering that IRF8 is a transcription factor, we used a transcription factor database JASPAR and predicted potential binding sites between IRF8 and the promoter region of BMAL1. Luciferase reporter assays showed that IRF8 transcription activates the expression of BMAL1 (Fig. 4E). qPCR and western blots analyses revealed that administration with AAV-IRF8 led to a significant up-regulation of hepatic BMAL1 expression compared with control mice, either under CD or HFD feeding conditions (Fig. 4F–I). In contrast, administration with AAV-shIRF8 down-regulated the expression of BMAL1 (Fig. 4J, K). These data indicate that IRF8 positively regulates BMAL1 expression in hepatocytes.

### IRF8 facilitates lipid accumulation in hepatocytes by up-regulating BMAL1

**Figure 5:**
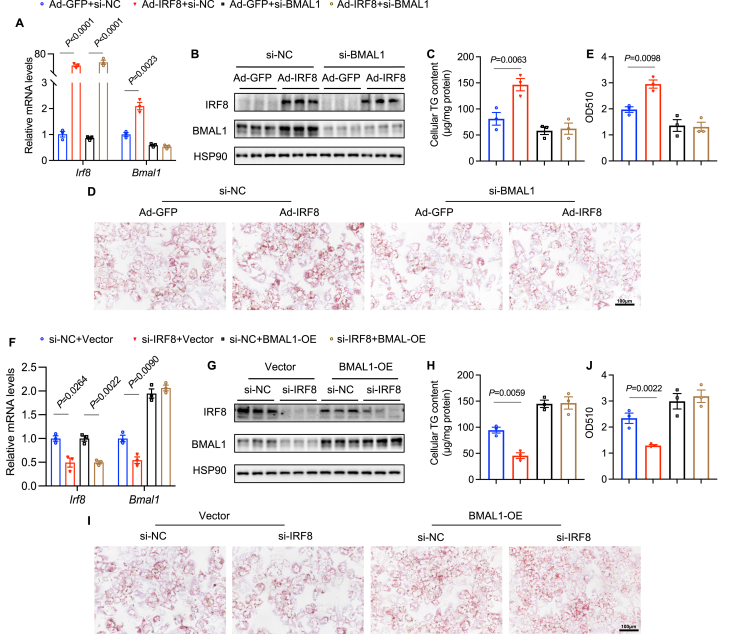
IRF8 facilitates lipid accumulation in hepatocytes by up-regulating BMAL1. **(A**–**E)** MPHs were transfected with either Ad-GFP or Ad-IRF8 to regulate the expression of IRF8, and with either si-NC or si-BMAL1 to control the expression of BMAL1, followed by treatment with 0.1 mM palmitic acid (PA) for 24 h. (A) At 6 h post-transfection, relative mRNA levels of *Irf8* and *Bmal1* were quantified using quantitative PCR. (B) At 48 h post-transfection, the protein levels of IRF8 and BMAL1 were analyzed by western blots. (C) TG content in MPHs, (D) Oil Red O staining, and (E) quantification of eluted Oil Red O (*n* = 3 biologically independent samples); scale bar: 100 μm. **(F–I)** MPHs were transfected with either si-NC or si-IRF8 to regulate IRF8 expression, and with either an empty vector or BMAL1 overexpression (OE) vector to control BMAL1 expression, with 0.1 mM PA treatment for 24 h. (F) At 6 h post-transfection, the relative mRNA levels of *Irf8* and *Bmal1* were measured using quantitative PCR. (G) At 48 h post-transfection, protein expression levels of IRF8 and BMAL1 were detected via western blots. (H) TG content in MPHs, (I) Oil Red O staining, and (J) quantification of eluted Oil Red O (*n* = 3 biologically independent samples); scale bar: 100 μm. Data are represented as mean ± standard error of the mean. *p* values were calculated using an unpaired two-tailed Student's *t*-test (A, C, E, F, H, J). IRF8, interferon regulatory factor 8; BMAL1, brain and muscle ARNT-like 1; MPHs, mouse primary hepatocytes; TG, triglyceride.

To further validate whether IRF8 regulates lipid metabolism through BMAL1, we overexpressed IRF8 in primary hepatocytes using Ad-IRF8 and knocked down BMAL1 using si-BMAL1. qPCR and western blots confirmed the up-regulation of IRF8 and the down-regulation of BMAL1 (Fig. 5A, B). Quantitative measurement of cellular TG showed that BMAL1 knockdown completely abolished the lipogenic effects induced by IRF8 overexpression in MPHs treated with palmitic acid (Fig. 5C). Similarly, Oil Red O staining of MPHs supported the reversal of lipogenesis mediated by IRF8 overexpression after BMAL1 silencing (Fig. 5D, E). Conversely, si-IRF8 treatment reduced IRF8 levels compared with si-NC, and overexpression of BMAL1 increased BMAL1 levels compared with the vector control, as confirmed by western blots and qPCR (Fig. 5F, G). Quantitative measurement of cellular TG and Oil Red O staining demonstrated that reduced IRF8 levels decreased lipid accumulation, which was counteracted by the increase in BMAL1 (Fig. 5H–J). Thus, these *in vitro* experiments indicate that IRF8 positively regulates BMAL1 transcription to promote lipid accumulation in hepatocytes.

### IRF8 regulates hepatic lipid metabolism via BMAL1/PPARγ axis

**Figure 6:**
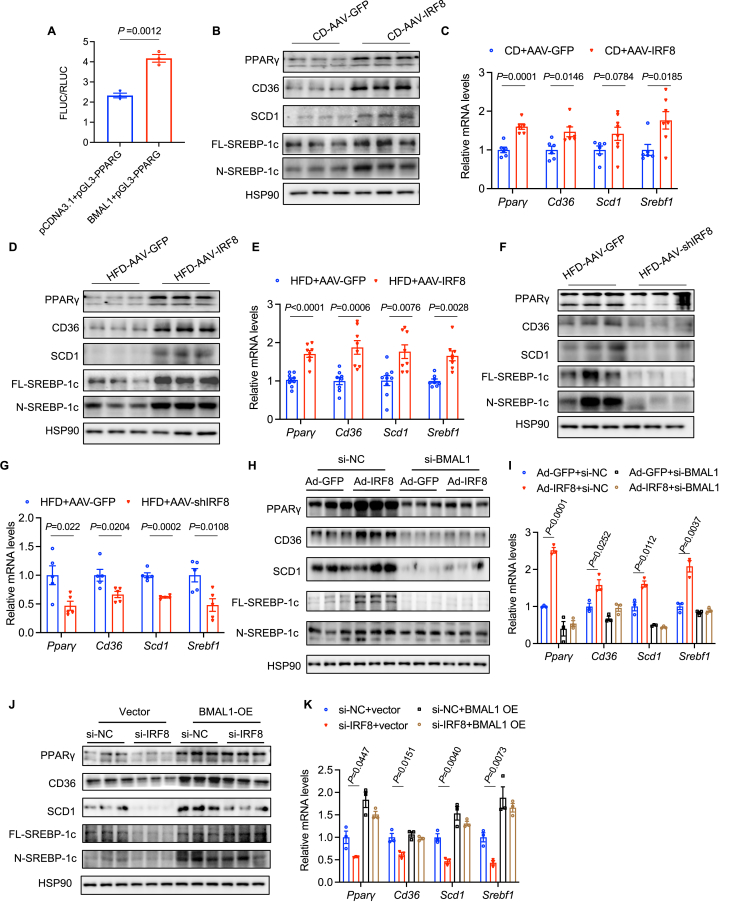
IRF8 regulates hepatic lipid metabolism via the BMAL1/PPARγ axis. **(A)** HEK293T cells co-transfected with *Pparγ* promoter reporter (pGL3-*Pparγ*), either pCDNA3.1 or BAML1 overexpression plasmid, were analyzed for relative luciferase activity (FLUC/RLUC) (*n* = 3 biologically independent samples). **(B**–**G)** Protein levels (*n* = 3 biologically independent mice from two groups) and relative mRNA levels of *Pparγ* and its target genes were assessed in the livers from (B, C) C57BL/6J male mice treated with AAV-IRF8 or AAV-GFP fed on CD (*n* = 6 biologically independent mice from two groups), and (D, E) C57BL/6J male mice treated with AAV-IRF8 or AAV-GFP fed on HFD (*n* = 8 biologically independent mice from two groups), and (F, G) C57BL/6J mice treated with AAV-shIRF8 or AAV-GFP fed on HFD. **(H, I)** Protein levels (H) and relative mRNA expression (I) of *Pparγ* and its target genes (*n* = 5 biologically independent mice from two groups) were analyzed in MPHs after transfection with combinations of Ad-GFP + si-NC, Ad-IRF8 + si-NC, Ad-GFP + si-BMAL1, and Ad-IRF8 + si-BMAL1, in the presence of 0.1 mM palmitic acid (PA) treatment (*n* = 3 biologically independent samples). **(J, K)** Protein levels (J) and relative mRNA expression (K) of *Pparγ* and its target genes were determined in MPHs transfected with si-NC + Vector, si-IRF8 + Vector, si-NC + BMAL1 OE, and si-IRF8 + BMAL1 OE, concurrently with 0.1 mM PA treatment (*n* = 3 biologically independent samples). Data are presented as mean ± standard error of the mean. *p* values were calculated using an unpaired two-tailed Student's *t*-test (B, D, F, H, I, J). IRF8, interferon regulatory factor 8; BMAL1, brain and muscle ARNT-like 1; PPARγ, peroxisome proliferator-activated receptor γ; HFD, high-fat diet; CD, chow diet; AAV, adeno-associated virus; GFP, green fluorescent protein; MPHs, mouse primary hepatocytes.

BMAL1 has been found to regulate various lipid metabolism genes. For instance, BMAL1 directly activates *Dgat2* (encoding diacylglycerol acyltransferase 2) by binding to the E-box in its promoter. Previous literature reported that BMAL1 deletion inhibits the expression of CD36 and peroxisome proliferator-activated receptor γ (PPARγ) and attenuates hepatic steatosis.^25^ In this study, KEGG analysis of the differentially expressed genes identified by RNA sequencing demonstrated that the PPAR pathway was significantly enriched. Based on the above literature search and RNA sequencing data analysis, we postulated that BMAL1 may regulate hepatic lipid metabolism through PPARγ. To validate our assumption, we then conducted predictions using the JASPAR database and found potential binding sites of BMAL1 within the promoter region of PPARγ. Similarly, luciferase reporter gene assays confirmed that BMAL1 positively regulates PPARγ expression in HEK293T cell lines (Fig. 6A). Western blots and qPCR analysis revealed that the expression of PPARγ and its target genes, including CD36, SCD1, and SREBF1, is up-regulated in the liver of mice transduced with AAV-IRF8, both in CD- and HFD-fed mice (Fig. 6B–E). In contrast, AAV silencing displayed opposite results in mice fed with HFD (Fig. 6F, G). These findings suggest that IRF8 promotes the expression of PPARγ and its downstream lipid metabolism genes through transcriptional activation of BMAL1. Similarly, transfection with Ad-IRF8 increased the expression of PPARγ, CD36, SCD1, and SREBP-1c in PA-treated MPHs when compared with Ad-GFP, which was nullified following co-transfection with si-BMAL1 (Fig. 6H, I). Conversely, si-IRF8 transfection led to reduced expression of PPARγ, CD36, SCD1, and SREBP-1c compared with the Ad-GFP group, yet co-transfection with a BMAL1 overexpression vector diminished these differences (Fig. 6J, K). *In vitro* and *in vivo* studies collectively indicate that IRF8 modulates the expression of PPARγ and related fatty acid uptake and synthesis genes mediated by BMAL1.

### PPARγ antagonist GW9662 abolishes the promotive effect of IRF8 on lipid accumulation in hepatocytes

**Figure 7:**
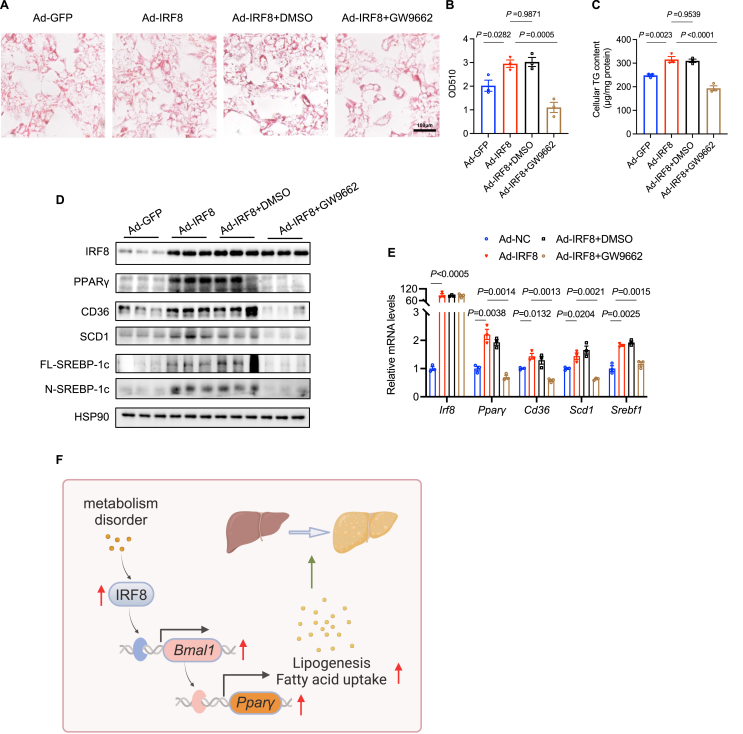
PPARγ antagonist GW9662 abolishes the promotive effect of IRF8 on lipid accumulation in hepatocytes. MPHs were transfected with Ad-GFP or Ad-IRF8 to modulate IRF8 expression and were treated with GW9662 (10 μM) to inhibit PPARγ, followed by treatment with 0.1 mM palmitic acid (PA) for 24 h. **(A, B)** At 48 h post-transfection, MPHs were subjected to Oil Red O staining (A), and quantification of eluted Oil Red O was performed (B). Scale bar: 100 μm. **(C)** The TG content of MPHs was measured. **(D)** The protein levels of IRF8 and PPARγ and its target genes were detected using western blots. **(E)** Relative mRNA levels of *Irf8* and *Pparγ* and its target genes were quantified by quantitative PCR (*n* = 3 biologically independent samples). **(F)** The schematic overview was constructed by Biorender (https://app.biorender.com). Data are presented as mean ± standard error of the mean. *p* values were calculated using an unpaired two-tailed Student's *t*-test (B, C, E, F). PPARγ, peroxisome proliferator-activated receptor γ; IRF8, interferon regulatory factor 8; TG, triglyceride; MPHs, mouse primary hepatocytes.

To further validate whether IRF8 promotes hepatic lipid accumulation through BMAL1-mediated changes in PPARγ. MPHs were transfected with Ad-IRF8 and treated with GW9662 (PPARγ inhibitor) or DMSO (vehicle) as a control under palmitic acid-treated conditions. Oil Red O staining (Fig. 7A, B) and quantitative measurement of cellular TG (Fig. 7C) demonstrated that increased IRF8 expression enhanced lipid accumulation in hepatocytes, and GW9662 treatment markedly reserved this effect. At the molecular level, IRF8 overexpression significantly induced the expression of PPARγ and its target genes, which was reversed by GW9662 treatment (Fig. 7D, E). These findings collectively indicate that IRF8 facilitates lipogenesis in hepatocytes via up-regulation of PPARγ signaling; suppression of PPARγ by the small molecule inhibitor GW9662 alleviates this effect.

## Discussion

The pathogenesis of NAFLD is a complex process involving multiple mechanisms. Among these, the mobilization of fatty acids leading to ectopic deposition of liver fat is considered a prominent mechanism. This process triggers endoplasmic reticulum stress, mitochondrial dysfunction, oxidative stress, increased cytokine production, and activation of hepatic stellate cells, ultimately contributing to liver inflammation and fibrosis.^26^, ^27^, ^28^ In this study, we investigated the role of IRF8 in regulating hepatic lipid metabolism. We observed elevated expression of IRF8 in NAFLD livers, suggesting its potential involvement in NAFLD. Further analysis revealed that IRF8 activated the transcription of BMAL1 and led to an increase in PPARγ levels, promoting hepatic lipid accumulation and contributing to the development of NAFLD. Correspondingly, *in vivo* AAV-mediated IRF8 knockdown in mice or *in vitro* Ad-mediated IRF8 silencing rescued the above metabolic disorder. Additionally, applying a PPARγ inhibitor sufficiently reversed the enhanced lipogenesis induced by IRF8. This study uncovers a critical function of IRF8 in hepatic lipid homeostasis. It sheds light on the underlying mechanism of NAFLD.

To our knowledge, this study is the first to reveal the lipid-modulating function of IRF8. IRF8 is a member of the interferon regulatory factor family, while the family members traditionally play crucial roles in regulating the interferon signaling pathway for innate immune response in myeloid and dendritic cells.^29^ Emerging evidence has expanded our understanding of their involvement in metabolic regulation. For instance, IRF9 has been reported to promote the transcription of *Pparα*, improving hepatic steatosis or inhibiting SIRT1 activity, thus mitigating liver ischemic injury.^30^ Additionally, IL-21-dependent IRF4 has been implicated in affecting adipose tissue Tregs and systemic insulin sensitivity.^31^ Moreover, IRF3 has been found to activate hepatic injury through the STING-IRF3 pathway,^32^ trigger endothelial inflammation induced by free fatty acids,^33^ and participate in β-cell lipotoxicity in type 2 diabetes.^34^ In this study, we provide direct evidence that IRF8 overexpression exhibited obvious lipid accumulation in the liver even under CD-feeding conditions. Moreover, the metabolic phenotypes were aggravated when IRF8 overexpression mice were on an HFD. These results demonstrate that elevation of hepatic IRF8 robustly induces hepatic steatosis.

The complex CLOCK:BMAL1 serves as the master regulator of the circadian clock.^35^ BMAL1-dependent transcription is intricately regulated to govern lipid handling. At the molecular level, mammalian CRY proteins act as the primary transcriptional repressor of CLOCK:BMAL1.^36^ In the liver, hepatocyte nuclear factor 4A strongly represses the transcriptional activity of the CLOCK:BMAL1 heterodimer. Additionally, an HFD triggers extensive remodeling of the liver clock, leading to impaired CLOCK:BMAL1 chromatin recruitment.^37^ Epigenetic mediators have been shown to influence BMAL1 expression,^38^ while microRNA and long non-coding RNAs have been reported to modulate CLOCK:BMAL1 expression.^39^^,^^40^ In this study, we demonstrate that IRF8 acts as an unreported BMAL1 transcription factor, influencing its promoter activity and thereby regulating BMAL1 expression in the liver. We have elucidated a previously unknown mechanism involving IRF8 in hepatic lipid metabolism, showing how IRF8 regulates fatty acid uptake and synthesis in a BMAL1-dependent manner.

BMAL1 is a pivotal regulatory element of the circadian rhythm. Functioning as a transcription factor, it forms a heterodimeric complex with CLOCK, facilitating the activation of downstream circadian gene expression. Increasing evidence has established a link between circadian genes and conditions such as obesity, type 2 diabetes, and NAFLD.^41^ Mice lacking BMAL1 exhibit disruptions in plasma TG daily rhythmicity,^42^ as well as reduced expression of several key lipogenic factors, such as PPARγ, adipocyte fatty acid-binding protein 2, CCAAT/enhancer-binding protein α, SREBP-1a, and fatty acid synthase.^43^ BMAL1 ablation inhibits the expression of CD36 and PPARγ signaling and attenuates hepatic steatosis.^25^ In this study, we observed an up-regulation of BMAL1 in hepatocyte-specific IRF8 overexpression mice fed both on a chow diet or an HFD. Conversely, the absence of IRF8 decreased BMAL1 levels in the hepatocytes. Notably, BMAL1 knockdown abolished the lipid accumulation induced by IRF8 overexpression. Here, we demonstrate that IRF8 acts as an unreported BMAL1 transcription factor, affecting its promoter activity, and thus regulating the expression of BMAL1 in the liver. We clarified previously unknown IRF8 involved hepatic lipid metabolism mechanism that IRF8 modulates *de novo* lipogenesis via BMAL1. These findings shed light on the intricate molecular mechanisms underlying NAFLD development, providing insights into potential therapeutic targets for this prevalent liver disorder.

PPARγ is a member of the nuclear hormone receptor superfamily and plays a significant role in modulating adipogenesis, glucose homeostasis, and inflammation. Extensive studies have substantiated that hepatic PPARγ induces the expression of lipid synthesis genes, thereby promoting hepatic lipid accumulation.^44^, ^45^, ^46^ Paradoxically, the clinical administration of PPARγ agonists for either short- or long-term treatment can ameliorate NAFLD.^47^ This discrepancy has been attributed to the predominant expression of PPARγ in adipose tissues, where systemic use of PPARγ agonists primarily elicits insulin-sensitizing effects that surpass their capacity to promote hepatic lipid accumulation.^48^, ^49^, ^50^ Consequently, decreased hepatic PPARγ expression specifically attenuates hepatic lipid accumulation. Our data demonstrate that IRF8 modulates PPARγ expression via a BMAL1-dependent manner, BMAL1 positively regulates the expression of PPARγ, thus promoting lipogenesis in the hepatocytes. IRF8 overexpression induced PPARγ, while IRF8 knockdown reduced its expression in both *in vivo* mouse models and *in vitro* primary hepatocytes. Notably, the application of a PPARγ inhibitor (GW9662) to IRF8-overexpressing MPHs effectively suppresses the cellular lipid deposition caused by IRF8 up-regulation. Our findings elucidate that the IRF8/BMAL1/PPARγ axis may play an important role in liver lipid metabolism (Fig. 7F).

Further studies will be required to investigate the precise mechanisms that up-regulate hepatic IRF8 in NAFLD models. It remains unclear whether IRF8 contributes to non-alcoholic steatohepatitis, the advanced stage of NAFLD. In addition, we reported here that IRF8 modulates hepatic lipid homeostasis by influencing genes associated with circadian rhythm; however, whether IRF8 leads to disturbances in the circadian rhythm needs further exploration.

In conclusion, this study reveals a previously unknown and crucial factor modulating hepatic lipid homeostasis and the mechanism of hepatic steatosis in NAFLD models. We identified IRF8 as a critical regulator involved in NAFLD progression. IRF8 may be a novel potential therapeutic target for NAFLD.

## Ethics declaration

All participants involved in the study signed informed consent forms. All procedures complied with the Helsinki Declaration and were approved by the Ethics Committee of the Capital Medical University Affiliated Shijitan Hospital [ethics approval number: sjtky11-1x-2022(098)]. All animal experimental procedures were approved by the Ethics Committee of the Sixth People's Hospital Affiliated to the Shanghai Jiao Tong University School of Medicine.

## CRediT authorship contribution statement

C.H., Y.H., L.W., and X.L., conceived, designed, and supervised the project. X.L. designed and performed essential experiments, analyzed data, and wrote the initial manuscript draft. H.Z. performed the analysis of RNA sequencing data. F.Y., S.X., and T.W. performed essential experiments. G.X. and R.Z. collected human samples.

## Conflict of interests

The authors declared no conflict of interests.

## Funding

This research was supported by grants from the 10.13039/501100001809National Science Foundation of China (No. 81974118, 82325010), The Shanghai Outstanding Academic Leaders (China) (No. 20XD1433300), the Shuguang Project of China (21SG11), the Innovative Research Team of High-level Local Universities in Shanghai, China (No. SHSMU-ZDCX20212700), the Major Natural Science Project of the Scientific Research and Innovation Plan of Shanghai Municipal Commission of Education (China) (No. 2023ZKZD17), the Shanghai Research Center for Endocrine and Metabolic Diseases (China) (No. 2022ZZ01002), and the Shanghai Sixth People's Hospital Foundation (China) (No. ynqn202105).
