# Supplementary material for: IRF8 aggravates nonalcoholic fatty liver disease via BMAL1/PPARγ axis
Source: Genes Dis. 2024 May 20;12(3):101333. doi: 10.1016/j.gendis.2024.101333 (PMC11905893; doi:10.1016/j.gendis.2024.101333)
Supplement: Multimedia component 1 [file mmc1.pdf]

Table. S1 The sequences of shRNA targeting IRF8.

Viruses	sequences
AAV-GFP	GAAGTCGTGAGAAGTAGAA
AV-shIRF8	GCCATATAAAGTTTACCGAAT

Table. S2 The sequences of si-RNA.

si-RNA	Forward sequence	Reverse sequence
si-NC	UUCUCCGAACGUGUCACGUTT	ACGUGACACGUUCGGAGAATT
si-IRF8	CUAUGACACACACCAUUCATT	UGAAUGGUGUGUGUCAUAGTT
si-BMAL1	GCCUCUACCUGUUCAAAGATT	UCUUUGAACAGGUAGAGGCTT

Table. S3 The primer sequences for RT-qPCR

Gene	Forward sequence	Reverse sequence
Irf8	CAATCAGGAGGTGGATGCTTCC	GTTCAGAGCACAGCGTAACCTC
Bmal1	ACCTCGCAGAATGTCACAGGCA	CTGAACCATCGACTTCGTAGCG
PPARγ	GTACTGTCGGTTTCAGAAGTGCC	ATCTCCGCCAACAGCTTCTCCT
Cd36	GGACATTGAGATTCTTTTCCTCTG	GCAAAGGCATTGGCTGGAAGAAC
Srebf1	CGACTACATCCGCTTCTTGCAG	CCTCCATAGACACATCTGTGCC
Scd1	GCAAGCTCTACACCTGCCTCTT	CGTGCCTTGTAAGTTCTGTGGC
Clock	GGCTGAAAGACGGCGAGAACTT	GTGCTTCCTTGAGACTCACTGTG
Actb	CATTGCTGACAGGATGCAGAAGG	TGCTGGAAGGTGGACAGTGAGG
